# The ethanolic extract of *Salvia lachnostachys* Benth is not maternotoxic, does not alter reproductive performance, but has teratogenic potential

**DOI:** 10.1186/s12906-023-03953-6

**Published:** 2023-05-04

**Authors:** Hudman Cunha Ortiz, Silvia Cordeiro das Neves, Cândida Aparecida Leite Kassuya, Henrique Rodrigues Scherer Coelho, Allana C. F. Martins, Marcelo Luiz Brandão Vilela, Valter Aragão do Nascimento, Arunachalam Karuppusamy, Maria Élida Alves Stefanello, Rodrigo Juliano Oliveira, Roberto da Silva Gomes

**Affiliations:** 1 Centro de Estudos Em Células TroncoTerapia Celular E Genética Toxicológica (CeTroGen), Faculdade de Medicina (FAMED), Federal University of Mato Grosso Do Sul (UFMS), Campo Grande, Mato Grosso Do Sul Brazil; 2 Programa de Pós-Graduação Em Saúde E Desenvolvimento Na Região Centro-OesteFaculdade de Medicina (FAMED), Federal University of Mato Grosso Do Sul (UFMS), Campo Grande, Mato Grosso Do Sul Brazil; 3 Faculdade de Ciências da Saúde (FCS), Universidade Federal da Grande Dourados (UFGD), Dourados, Mato Grosso Do Sul Brazil; 4 Department of Pharmaceutical Sciences, North Dakota State University, Fargo, ND 58102 USA; 5 Faculdade de Medicina (FAMED), Federal University of Mato Grosso Do Sul (UFMS), Campo Grande, Mato Grosso Do Sul Brazil; 6 Group of Spectroscopy and Bioinformatics Applied to Biodiversity and Health (GEBABS), Graduate Program in Health and Development in the Central-West Region of Brazil, School of Medicine, Federal University of Mato Grosso do Sul, Campo Grande, Brazil; 7 Departamento de Química, Universidade Federal Do Paraná (UFPR), Curitiba, Paraná Brazil

**Keywords:** Teratogenesis, Malformation, Genotoxicity, Medicinal plants

## Abstract

*Salvia lachnostachys Benth* is native to Brazil and has anti-inflammatory, anti-arthritic, cytotoxic, antitumor, and antihyperalgesic activities. The population, including pregnant women, consume this plant to treat pain, inflammation, flu, spasms, insomnia, and depression, mainly. There are no safety reports on the use of this plant during pregnancy. The present study aimed to evaluate the effects of *S. lachnostachys* ethanolic extract (EESl) on reproductive performance, embryofetal development, and DNA integrity of pregnant female mice. Pregnant females were randomly divided into three experimental groups (*n* = 10): The Control group was treated with a vehicle, and treatment groups were administered with EESl at 100 and 1000 mg/kg, respectively. Treatment occurred by gavage throughout the gestational period until day 18. Afterward, reproductive performance, embryofetal development, and DNA integrity parameters were evaluated. The results indicated that EESl did not alter any reproductive performance parameters. However, it changed embryofetal outcome through reduced placental weight (EESl 100 mg/kg), decreased fetal weight (EESl 100 and 1000 mg/kg), and increased frequency of small for gestational age fetuses (EESl 1000 mg/kg). In addition, EES1 increased the frequency of external, visceral, and skeletal malformations. Because of the above, it is considered that EESl is not maternotoxic, does not alter reproductive performance, but does alter embryofetal development. Its use in the gestational period is not indicated due to its teratogenic potential.

## Introduction

The species, *Salvia lachnostachys* Benth, is an endemic perennial herb of Brazil, occurring wild only in the South and Southeast regions of the country [1]. It is considered an ornamental species [2] with the possibility of being distributed to other parts of the country. This plant is known as “melissa” and has been used for treating spasms, flu, and insomnia in popular medicine [3].

The essential oil from the flowers and leaves of *S. lachnostachys* has saturated aliphatic compounds and a sesquiterpene fraction [4]. Diterpenes, triterpenes, and rosmarinic acid were isolated from the ethanolic extract of the leaves [5, 6]. The chromatographic profile of the ethanolic extract also was obtained by HPLC [7].

The ethanolic extract of *S. lachnostachys* has already been described with anti-inflammatory, cytotoxic, anti-arthritic, analgesic, antidepressant, and antinociceptive activities. Fructiculin A (a diterpene isolated or derived from *S. lachnostachys*) is believed to be one of the compounds that assist in these properties, and is cited as anti-inflammatory, antihyperalgesic, antidepressant and antinociceptive [5, 8-10]. Furthermore, in the literature, *S. lachnostachys* extract was characterized as chemopreventive and antitumor in Ehrlich models [7] and fruticulin A as antineoplastic in vitro and in vivo models [11]. Four diterpenes (fructiculine A, demethylfruticuline A, fruticuline B, and demethylfruticuline B) showed antioxidant activity [6].

In a recent publication, we reported the characterization of *S. lachnostachys* extract. We identified the presence of three new diterpenoids: demethylfruticuline B, 20-hydroxyfruticuline B, and 6-hydroxyisofruticuline A, isolated from the leaves of *S. lachnostachys*. We also reported the presence of five known compounds: fruticuline B, fruticuline A, demethylfruticuline A, heterobetulinic acid, and maslinic acid [6].

Considering the above, it appears that *S. lachnostachys* extract can primarily be used to treat inflammatory processes, pain, and depression. Pregnant women are affected by all these conditions. Anti-inflammatory and analgesic drugs are widely used during pregnancy, as pointed out by studies conducted by Fontoura et al. [12] and Lima et al. [13]. Depression is also common during pregnancy and the use of antidepressants in this period has increased significantly in the last 20 years [14]. As an example, we can mention duloxetine, which can lead to an increase in the number of abortions [15], and selective serotonin reuptake inhibitors, which increase the risk of congenital heart diseases, neonatal maladaptive syndrome, or persistent pulmonary hypertension. This is because serotonin is essential for developing all embryonic cells and organogenesis [14, 16-18]. In view of the above, we consider that allopathic drugs can harm embryonic and fetal development. In this way, many pregnant women seek natural alternatives for the treatment of pain, inflammation, flu, spasms, insomnia and depression, mainly through the use of *S. lachnostachys* tea. There are no eport regarding the safety on the use of this plant during pregnancy. However, reports of people who are using this tea are growing. Thus, there is a need to evaluate the safety of using this product during the gestational period, since in locations distant from large centers, where populations make use of teas for the primary treatment of these conditions, exposure of pregnant women to *S. lachnostachys* tea would not be uncommon. Thus, the present research aimed to evaluate the effects of the ethanolic extract of *S. lachnostachys* (EESl) on reproductive performance, embryofetal development, and DNA integrity of pregnant female mice.

## Materials and methods

### Plant material and extract preparation

Leaves of *S. lachnostachys* were collected in Curitiba, Paraná, Brazil (25°30′44,6"S, 49°18′7,13"W) from a natural population in May 2010. E. P. Santos identified the plant, and a voucher specimen was deposited in the Herbarium of the Universidade Federal do Paraná (UPCB 85,285). The collection followed the Brazilian National guidelines. The approved code registered in the National System for the Management of Genetic Heritage and Associated Traditional Knowledge (SISGEN/Brazil) is A19F875. Leaves were dried at 40° in an oven for seven days. Then, the dried material (415.3 g) was powdered in a mill, and extracted with hexane followed by ethanol (3 × 2 L, for each solvent). The solvents were removed under reduced pressure, yielding the respective extracts in hexane (2.7%) and ethanol (EESl, 11.5%). The preliminary extraction with hexane removes long-chain aliphatic compounds (“waxes”) that are abundant in leaves, in order to facilitate the isolation of secondary metabolites. Waxes do not present biological activities. The ethanolic extract was maintened in a freezer (at -4° C) until the tests were performed. Thin layer chromatography (TLC) analyses showed no alteration after the time of storage. The ethanol extract has a high content of rosmarinic acid [7], which is an antioxidant and contribute for its stability. Pharmacological tests were performed with crude ethanolic extract of S. lachnostachys leaves (EESl) diluted in saline.

### Animals

The animals were provided by the Central Animal Facility of the Federal University of Mato Grosso do Sul in a total of 60 mice (*Mus musculus*) of the *Swiss* strain of both sexes at reproductive age: 30 females with an average weight of 30 g and 15 males with an average weight of 35 g. All animals underwent a 7-day adaptation period and were placed in mini-isolators (Alesco® ventilated rack) lined with sawdust (*Pinus* sp.). The males were isolated, and the females were in pairs. The animals were kept under temperature control (22 ± 2 °C), light by photoperiod (12 h light/ 12 h dark), relative humidity of 55 ± 10%, and access to feed and water ad libitum. The research was conducted according to the Universal Declaration of Animal Rights protocols and with approval from the Ethics Committee on Animal Use (CEUA) of the Federal University of Mato Grosso do Sul (UFMS) under opinion number 965/2018.

### Experimental design

The animals were mated during the night at a ratio of 1 male: to 2 females. Females were not subjected to estrous cycle synchronization. All females were placed in mini-isolators in contact with males every day at 6 pm and were removed the following day at 6 am for pregnancy assessment. The detection of vaginal plugs in females determined pregnancy occurrence, and this day was considered day zero of gestation [19-21]. Pregnant females did not return to the mini-isolators with the males. However, non-pregnant females went through new matings until all females became pregnant (this period did not exceed 2 weeks [21]. Pregnant females were randomly divided into three experimental groups (*n* = 10): Control Group—animals received 0.1 mL/10 g body weight (b.w.) of vehicle (in saline – 0.9% NaCl) by gavage (p.o.) throughout gestation (gestational day 1 to 18); EESl Groups—animals received EESl by gavage at doses of 100 and 1000 mg/kg (b.w., p.o.) during gestation. Such doses were chosen based on the study by Radai et al. [10] and from the recommendations of guidelines in the field [21-23]. According to Radai et al. [10], the dose of 50 and 100 mg/kg has anti-arthritic and anti-inflammatory effects. Thus, it was decided to test the highest effective dose and a dose ten times greater, as recommended by the literature in the area, to ensure the safety of use by pregnant women. According to Wilson and Warkanny (1965) the pre-implantation period is from the 1^st^ to the 5^th^ gestational day [24]. Thus, we also wanted to evaluate possible pre-implantation losses, the treatments were started on the 1^st^ gestational day.

### Biological assays

Peripheral blood was collected by tail vein puncture on the 16^th^, 17^th^, and 18^th^ gestational days (g.d.). After the last blood collection, the females were euthanized by cervical dislocation, followed by laparotomy, omphalectomy, and hysterectomy for collection, weighing, and proper storage of organs, fetuses, and placentas.

**Fig. 1:**
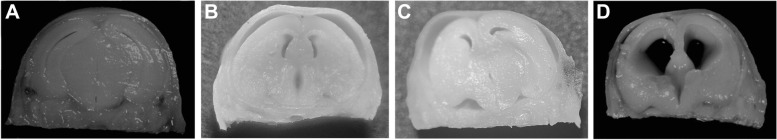
Photomicrograph of brain microdissections. **A **Normal brain (aspect of the lateral ventricles and the third and fourth ventricles). **B**-**D **Hydrocephalus—excessive cerebrospinal fluid within the sckull: **B**—mild grade; **C** – moderate grade and **D** – severe grade

**Fig. 2:**
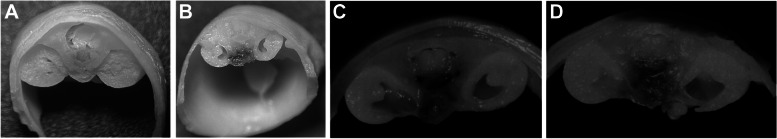
Photomicrograph of rim microdissections. **A **Rim (aspect of renal papilla and renal pelvis). **B**-**D **Hydronephrosis – marked dilatation of the renal pelvis and calyces secondary to obstruction of urine flow, usually combined with destruction of the renal parenchyma: **B** – gravity; **C** – moderate grain and **D** – severe grain

The fetuses underwent systematic analysis for external malformations and sexing and were then randomly distributed into two subgroups. The first subgroup was destined for visceral analysis. It was fixed in Bodians' solution, composed of distilled water (142 mL), acetic acid (50 mL), formaldehyde (50 mL), and 95% alcohol (758 mL), for at least seven days. They were then submitted to incision/microdissection with strategic cuts proposed by Barrow and Taylor [25] for the study of the thorax and abdomen and by Wilson [26], modified by Oliveira et al. [19] for the study of the head. The classification of visceral changes was based on the experiments described by Taylor [27], Manson and Kang [28], Wise et al. [29], Damasceno et al. [30], and Oliveira et al. [19]. Hydrocephalus and hydronephrosis were classified as mild, moderate or severe according to the pattern shown in Figs. 1 and 2. The second subgroup, intended for skeletal analysis, was fixed in absolute acetone for at least seven days. After removal of the viscera, the fetuses were immersed in KOH (0.8%) and stained with Alizarin Red during the diaphanization process, as proposed by Straples and Schenell [31] with modifications by Oliveira et al. [20]. After the fetuses were stained, the KOH solution was replaced with the bleaching solution (1 L glycerin: 1 L ethyl alcohol: 0.5 L benzyl alcohol), which was changed every 24 h for five days. The fetal viscera and skeletons analyses were performed under a stereomicroscopic magnifying glass (Nikon®—SMZ 745 T) at 1.6 × magnification.

### Evaluation of biological parameters

The biological parameters were calculated from the records of initial weight (females weighed at day zero), final weight (females weighed at 18 g.d.), weight gain (final weight—initial weight), uterus weight, net weight gain (weight gain—uterus weight), absolute and relative weights of heart, lung, spleen, kidneys, and liver.

### Reproductive performance and embryofetal development

The absolute number of implantation (number of live fetuses + number of dead fetuses + number of resorptions), resorption of embryos or fetuses in the uterus, live and dead fetuses was registered. The fetal and placental weigth (g) were mensured on a precision scale. Based on these data, we obtained: fetal viability (number of live fetuses × 100 / number of implantations), post-implantation loss rate [(number of implantations—number of resorptions) × 100 / number of implantations], resorption rate (number of resorptions X 100 / number of implantations), placental index (placental weight / fetal weight), placental efficiency (fetal weight / placental weight) and sex ratio (number of male fetuses/numbers of female fetuses).

The classification of fetal weight according to gestational age (CFWGA). After collecting fetuses from the uterine horns, they were weighed, and according to the mean ± 1.7 × standard deviation (SD) of the body weights obtained in the control group, they were classified as adequate for gestational age (AGA), small for gestational age (SGA) and large for gestational age (LGA).

### Evaluation of micronucleus in peripheral blood

Micronucleus testing in peripheral blood was performed according to the method described by Hayashi et al. [32] and modified by Oliveira et al. [20]. Peripheral blood, collected by tail vein puncture (about 20μL), was deposited on a slide prepared with Acridine Orange (1 mg/mL). The blood was covered with a coverslip, and the material was stored in a freezer (-20 °C) for a minimum of seven days. A total of 2,000 reticulocytes were examined per animal using an EVOS M7000 microscope (Zeiss®) at 40 × magnification with an excitation filter of 470 nm ± 22 and an emission filter of 525 nm ± 50. The frequency of micronuclei was calculated by the sum of micronuclei observed in 2,000 reticulocytes/animal divided by the sample *n* (*n* = 10 animals/group).

### Statistical analysis

Data distribution was evaluated using the Kolmogorov–Smirnov test. The normal distribution data for the one-way ANOVA test with Tukey post-test was used. For other data, the Kruskal–Wallis test with the Dunn post-test was used. For frequency comparisons, the Chi-square test was used. The analyses were performed using the GraphPad Instat® program (Version 3.06, 2003). Data were presented as mean ± standard error of the mean or mean ± standard deviation, and the established significance level was *p* < 0.05.

## Results

The initial weight, final weight, uterine weight, net weight gain, the absolute weight of the heart, lung, spleen, and kidneys, and the relative weight of the heart, lung, spleen, kidneys, and liver showed no statistically significant differences between the experimental groups (*p* > 0.05). However, there was a reduction (*p* < 0.05) in weight gain and a reduction in absolute liver weight by EESl at a dose of 100 mg/kg when compared to the control group (Table 1).

**Table 1:** Biological parameters, absolute weight, and relative weight of organs of females treated with the ethanolic extract of *S. lachnostachys* (EESl)

Biological parameters					
**Experimental groups (mg/kg)**	**Initial weight**^**2**^	**Final weight**^**1**^	**Weight gain**^**1**^	**Uterus Weight**^**1**^	**Net Weight gain**^**1**^
Absolute Weight (g)					
Control	29.26 ± 0.57^a^	53.94 ± 0.95^a^	24.67 ± 0.84^a^	19.71 ± 0.65^a^	4.97 ± 0.64^a^
EESl 100	29.46 ± 0.38^a^	49.66 ± 1.23^a^	20.20 ± 1.07^b^	16.97 ± 0.85^a^	3.23 ± 0.43^a^
EESl 1000	29.15 ± 0.73^a^	51.69 ± 1.66^a^	22.53 ± 1.36^ab^	18.40 ± 1.05^a^	4.13 ± 0.49^a^
Absolute Weight of organs (g)					
	Heart^2^	Lungs^1^	Spleen^2^	Kidney^2^	Liver^1^
Control	0.18 ± 0.01^a^	0.23 ± 0.01^a^	0.15 ± 0.01^a^	0.40 ± 0.02^a^	2.49 ± 0.05^a^
EESl 100	0.16 ± 0.00^a^	0.21 ± 0.01^a^	0.13 ± 0.01^a^	0.36 ± 0.02^a^	2.15 ± 0.08^b^
EESl 1000	0.17 ± 0.01^a^	0.23 ± 0.01^a^	0.14 ± 0.00^a^	0.38 ± 0.01^a^	2.42 ± 0.11^ab^
Relative Weight of organs					
	Heart^2^	Lungs^1^	Spleen^2^	Kidney^2^	Liver^1^
Control	0.003 ± 0.00^a^	0.004 ± 0.00^a^	0.003 ± 0.00^a^	0.01 ± 0.00^a^	0.05 ± 0.00^a^
EESl 100	0.003 ± 0.00^a^	0.004 ± 0.00^a^	0.003 ± 0.00^a^	0.01 ± 0.00^a^	0.04 ± 0.00^a^
EESl 1000	0.003 ± 0.00^a^	0.004 ± 0.00^a^	0.003 ± 0.00^a^	0.01 ± 0.00^a^	0.05 ± 0.00^a^

*Legend*: *g* grams. Equal letters indicate no statistically significant differences. Mean ± Standard error of the mean (1—Anova/Tukey’s test; 2—Kruskal–Wallis/Dunn, *p* > 0.05)

Treatment with EESl, at both doses, caused no statistically significant changes (*p* < 0.05) in the number of implants, live fetuses, dead fetuses, the average number of fetuses per litter, fetal viability, post-implantation loss rate, resorption, resorption rate, and sex ratio (Table 2).

**Table 2:** Reproductive parameters of females treated with the ethanolic extract of *S. lachnostachys* (EESl)

Reproductive performance			
**Experimental Groups (mg/kg)**			
**Parameters**	**Control**	**EESl 100**	**EESl 1000**
Implants^1^	13.50 ± 0.43^a^	12.30 ± 0.60^a^	13.89 ± 0.63^a^
Live Fetuses^1^	13.10 ± 0.41^a^	11.80 ± 0.61^a^	13.00 ± 0.71^a^
Dead Fetuses	0.00 ± 0.00^a^	0.00 ± 0.00^a^	0.00 ± 0.00^a^
Average no. of fetus^1^	13.10 ± 0.41^a^	11.80 ± 0.61^a^	13.00 ± 0.71^a^
Fetal Viability^2^	97.12 ± 1.18^a^	96.02 ± 1.75^a^	93.42 ± 1.84^a^
PILR^2^	02.88 ± 1.18^a^	03.97 ± 1.75^a^	06.58 ± 1.84^a^
Reabsorption^2^	0.40 ± 0.24^a^	0.50 ± 0.45^a^	2.12 ± 0.44^a^
Reabsorption Rate^2^	04.55 ± 0.16^a^	09.12 ± 0.22^a^	0.89 ± 0.26^a^
Sexual Reason^1^	161.54 ± 28.77^a^	141.00 ± 22.51^a^	123.48 ± 15.16^a^

*Legend*: *PILR* post-implantation loss rate; Equal letters indicate no statistically significant differences. Mean ± Standard Error of the mean (1—Anova/Tukey’s test; 2—Kruskal–Wallis/Dunn, *p* > 0.05)

A reduction (*p* < 0.05) in placental weight was observed in the EESl at a dose of 100 mg/kg and fetal weight reduction in the treatment of EESl at doses 100 and 1000 mg/kg groups. There was also an increase (*p* < 0.05) in the frequency of small for gestational age (SGA) fetuses in the EES1 1000 mg/kg group. The placental index did not differ from the control, but there was a reduction in the treatment of ESSl (*p* < 0.05) at a dose of 100 mg/kg when compared to the amount of 1000 mg/kg (Table 3).

**Table 3:** Parameters of embryofetal development and placental efficiency of females treated with the ethanolic extract of *S. lachnostachys* (EESl)

Embryo development and placental efficiency			
**Experimental groups (mg/kg)**			
**Parameter**	**Control**	**EESl 100**	**EESl 1000**
Placental Weight (g)^1^	0.0916 ± 0.00^a^	0.0816 ± 0.00^b^	0.0918 ± 0.01^a^
Placental Index^1^	0.0736 ± 0.00^ab^	0.0717 ± 0.00^a^	0.0776 ± 0.00^b^
Placental Efficiency^1^	14.3326 ± 0.29^ab^	14.9859 ± 0.36^a^	13.5319 ± 0.27^b^
Fetal Weight (g)^1^	1.2572 ± 0.01^a^	1.1584 ± 0.01^b^	1.1903 ± 0.01^c^
% SGA^2^	7.6923	16.2393	18.2608*
% AGA^2^	89.2307	83.7606	80.8695
%LGA^2^	3.0769	00.00	0.8695

*Legend*: *g* grams, *SGA* small for gestational age, *AGA* adequate for gestational age, *LGA* large for gestational age; Statistical test: 1 Anova/Tukey—Mean ± Standard error of the mean: different letters indicate statistically significant differences; 2 Chi-square test; *indicates statistically significant difference compared to the control group; *p*˂0.05

The external malformations observed were curved tail, posterior paw hyperflexion (uni and bilateral), posterior paw hyperextension (uni and bilateral), unilateral anterior paw hyperextension and hyperflexion, gastroschisis, hyperlordosis, scoliosis, hematoma, and nasal cavity obstruction. An increase (*p* > 0.05) in the frequency of curved tail and hyperlordosis was observed in the EESl 1000 group and scoliosis in the EESl 100 and EESl 1000 groups. The frequency of malformations and the percentage of fetuses with malformations increased (*p* < 0.05) in the treatment of EESl at doses 100 and 1000 mg/kg groups (Table 4).

**Table 4:** External malformations found in the offspring of females treated with the ethanolic extract of *S. lachnostachys* (EESl)

External malformation			
**Experimental groups (mg/kg)**			
**Parameters**	**Control**	**EESl 100**	**EESl 1000**
Analyzed Fetuses	131	118	117
Normal Fetuses	116	86	73
Curved Tail	0	3	5*
UPPH	0	3	1
BPPH	2	0	0
UPHPH	5	11	13
BPHP	1	3	0
UAHP	2	0	1
UAHPH	1	0	0
Gastroschisis	1	0	0
Hyperlordosis	0	2	10*
Scoliosis	2	7*	7*
Hematoma	0	1	3
Nasal Septum Obstruction	1	0	0
Frequency of Malformation	15	30*	40*
%M.F	11.45%	25.42%*	34.19%*

*Legend*: *UPPH* Unilateral posterior hind paw hyperflexion, *BPPH* bilateral posterior hind paw hyperextension, *UPHPH* Unilateral posterior hind paw hyperextension, *BPHP* bilateral posterior of hyperextension of hind paw, *UAHP* unilateral anterior hyperextension of the hind paw, *UAHPH* Unilateral anterior hind paw hyperflexion; *% M.F*. the percentage of malformed fetuses

^*^Statistically significant difference (Statistical test: Chi-square; *indicates statistically significant difference compared to the Control group; *p* < 0.05)

Concerning visceral malformations, mild and moderate grades hydrocephalus (excessive cerebrospinal fluid within the sckull), mild hydronephrosis (marked dilatation of renal pelvis anda calices secondary ti obstruction of urine flow, usually combined with destruction of the renal parenchyma), and absence of right kidney were identified. An increase (*p* < 0.05) in the occurrence of mild hydronephrosis was observed in the EES1 100 group, which determined an increase (*p* < 0.05) in the frequency of malformations and the percentage of fetuses with a malformation in the EESl 100 group compared to the control group (Table 5).

**Table 5:** Visceral malformations found in the offspring of females treated with the ethanolic extract of S. lachnostachys (EESl)

Visceral Malformation			
**Experimental groups (mg/kg)**			
**Parameters**	**Control**	**EESl 100**	**EESl 1000**
Analyzed fetuses	55	53	53
Normal Fetuses	44	35	45
**Brain—Hydrocephalus**			
Mild Hydrocephalus	10	7	6
Moderate Hydrocephalus	1	2	0
Frequency of Malformations	11	9	6
% M.F	20	16.98	11.32
**Urogenital Region—Hydronephrosis**			
Mild Hydronephrosis	0	8*	1
Absence of Right Kidney	0	1	0
Frequency of Malformations	0	9*	1
% M.F	0	16.98*	1.88

*Legend*: % M. F—the percentage of fetuses with malformation

^*^Statistical difference (Statistical test: Chi-square; *p* < 0.05)

The skeletal malformations found were the absence and reduced number of anterior and posterior phalanges, reduced number of metacarpal and metatarsal bones, decreased ossification and absence of the last sternal center, lack of the sternum, reduced ossification of the nasal, parietal, interparietal, supraoccipital, volar, palate and frontal bones, and absence of the basiphoid and hamulus bones. There was an increase (*p* < 0.05) in the percentage of malformed fetuses in the treatment of EES1 at doses 100 and 1000 mg/kg when the sternum and skull were evaluated. There was also an increase (*p* < 0.05) in the frequency of malformation for both groups when the skull was assessed (Table 6).

**Table 6:** Skeletal malformations found in the offspring of females treated with the ethanolic extract of *S. lachnostachys* (EESl)

Skeletal Malformation				
**Experimental groups (mg/kg)**				
**Parameters**		**Control**	**EESl 100**	**EESl 1000**
Analyzed fetuses		45	44	55
Normal Fetuses		18	11	20
**Members**				
Anterior phalanges	Absence	0	2	0
	Reduced number	4	2	2
Posterior phalanges	Absence	0	2	0
	Reduced number	1	2	3
Freq.Malf		5	8	5
% M.F		11.11	18.18	9.10
MTC	Reduced number	0	1	0
MTT	Reduced number	0	1	0
Freq.Malf		0	2	0
%M.F		0	4.55	0
**Sternum**				
Sternum Bones	O.R. Last Center Sternal	19	22	33
	Absence of the Last Sternal Center	1	6	3
Absence		0	1	0
Freq.Malf		20	29	36
%M.F		44.44	65.90*	65.45*
**Cranium**				
Nasal	R.O	0	3	4
Parietal	R.O	0	3	3
Interparietal	R.O	0	3	3
Supraoccipital	R.O	0	1	3
Volmer	R.O	3	8	0
Palate	R.O	1	5	1
Basisphenoid	Absence	0	1	0
Hamate	Absence	0	1	0
Frontal	R.O	0	1	4
Freq.Malf		4	26*	18*
% M.F		8.89	59.09*	32.73*

Legend: *MTC* metacarpal, *MTT* metatarsal, *R.O* Reduced Ossification, *Freq.Malf* Frequency of Malformations, % F.M— the percentage of fetuses with malformation

^*^Statistical difference (Statistical test: Chi-square; *p* < 0.05)

**Fig. 3:**
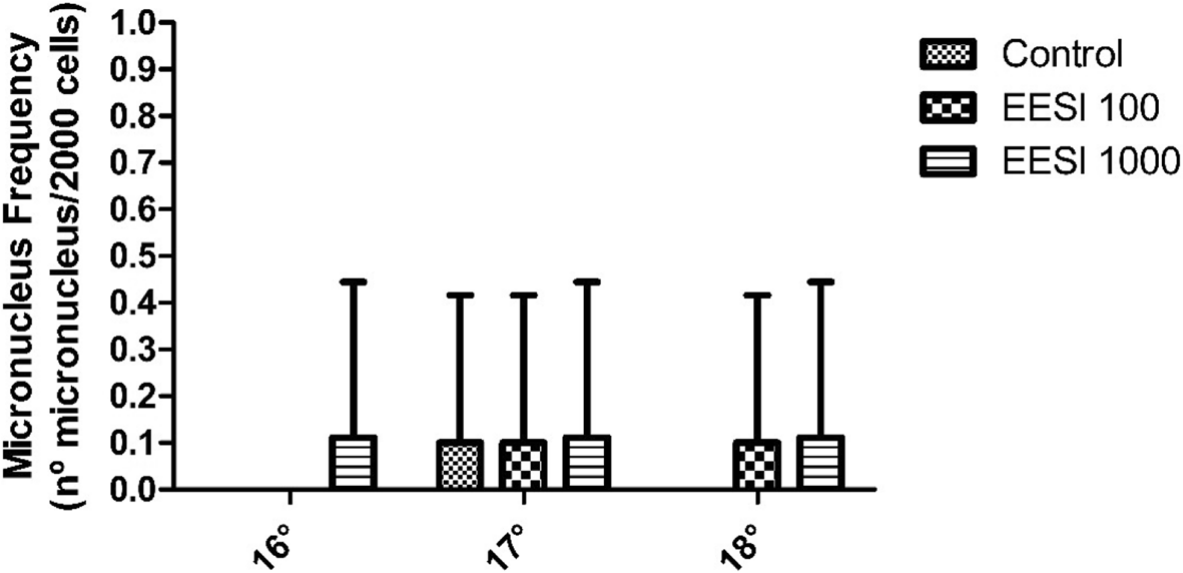
Frequency of micronuclei in females treated with the ethanolic extract *S. lachnostachys* (EESl). Statistical test: ANOVA/Tukey (*p* > 0.05); Mean ± Standard Deviation

The frequency of chromosomal damage did not vary among the different experimental groups, indicating that EESl does not alter the frequency of micronuclei (*p* > 0.05). There were also no significant variations between the various analysis times, meaning that EESl has no cumulative effect (*p* > 0.05) (Fig. 3).

## Discussion

According to the literature, no data on maternal toxicity, reproductive performance, and embryofetal development were found for *S. lachnostachys*. This demonstrates the importance, uniqueness, and pioneering nature of this study. Our findings will contribute to the ethnopharmacological indication and safety of the use of this plant since there are few studies of this species that deal with the biology of the plant and its medicinal properties [7-11].

Our results show no signs of maternotoxicity, such as diarrhea, tremors, excessive salivation, convulsions, hypoactivity, ataxia, lethargy, tail curvature and hair raising [7]. However, for the EES1 100 group, a significant decrease in weight gain and absolute liver weight was observed. According to Teng et al. [33], weight gain and organ weight variations can indicate toxicity. However, these findings in the present study do not appear to be associated with maternotoxicity, as there was no reduction in net weight gain. However, altered embryonic development and placental efficiency are suggested.

The animals started the experiments with similar weights; even EES1 at a dose of 100 mg/kg had the highest average. However, during gestation, this group showed the lowest weight gain. This fact was not only attributed to the lower number of fetuses since the difference in fetus frequency between the two EES1 treated groups was only one fetus. Additionally, the average fetal weight of EES1 at a dose of 100 mg/kg was the lowest of all the groups. Thus, these two facts may contribute to understanding weight gain reduction.

Therefore, it is assumed that these animals had a lower biometric development (lower maternal weight gain) without the influence of the treatment, i.e., without maternotoxicity. In this context, the weight of the organs (of the liver) may vary according to the animal's size (large animals have heavier livers than small animals). This inference can be corroborated by the absence of a significant difference in the relative weight of this same organ. The relative weight corrects distortions of this type since it normalizes the data by making a ratio between the organ's weight and the animal's final weight. Thus, as there are no significant differences in the relative weight, it is inferred that such data is an isolated occurrence and is not due to the treatment. It is also noteworthy that, in general, xenobiotics that cause damage to the body lead mainly to an increase in the liver and kidneys and not their decrease [34]. Therefore, our results suggest no maternal toxicity at 100 and 1000 mg/kg doses according to the present design.

The absence of maternotoxicity is the absence of genotoxic effects evaluated by the micronucleus test. Regulatory agencies internationally accept this test for chromosomal damage assessment [34, 35]. In our study, we used the collection of three peripheral blood samples over 72 h to verify whether EESl had a cumulative effect. Yet, there was an absence of chromosomal damage at all of the analyzed times, and thus no cumulative effects. These facts reinforce the idea that EESl does not cause maternal toxicity or genotoxicity at 100 and 1000 mg/kg doses.

This safety of use is corroborated by the studies of Radai et al. [10] and Corso et al. [7]. According to Radai et al. [10], EESl has no genotoxic effect in male Swiss mice treated with a dose of up to 1,000 mg/kg. However, this author reported the need for subacute and chronic toxicity testing, providing complementary responses to genotoxicity. Corso et al. [7] noted that EES1 extract showed no acute toxicity up to a dose of 2,000 mg/kg in Swiss females.

Evaluation of parameters related to reproductive performance indicated that there were no differences (*p* < 0.05) between the control and EESl-treated groups for the number of implantations, number of resorptions, resorption rate, number of live and dead fetuses, the mean number of fetuses, post-implantation loss rate, fetal viability, or sex ratio. These findings suggest that EESl does not interfere with female reproductive performance and does not cause any embryofetal lethality.

According to David et al. [36] and Gonçalves et al. [37], the absence of change in these parameters indicates the safety of the extract and no interference with reproductive performance. When evaluating embryofetal development, a reduction in fetal weight was observed for the groups treated with EES1. However, the placental index and efficiency did not vary compared to the control group. It was also observed that the lowest placental index occurred in EESl (100 mg/kg) and the lowest placental efficiency in the EESl 1000 (mg/kg) group. Given these results, it is suggested that the EESl (100 mg/kg) group had the lowest placental average and the lowest placental index (even though it was not different from the control group). The lowest fetal weight averages were observed when determining the lowest embryofetal growth for this group. This fact occurred even in the presence of the higher placental efficiency (which did not differ from the control group but was higher than the placental efficiency observed for the EESl (1000 mg/kg).

Thus, it is considered that even in the face of reorganization of the organism to increase placental efficiency, the compensation of the EES1 (100 mg/kg) was insufficient to guarantee the same embryofetal growth in the mean fetal weight observed in the other experimental groups. However, when weight adequacy for gestational age was performed, it was observed that there were no significant differences between the fetuses of the treatment of EESl at a dose of 100 mg/kg and the control group, i.e., the occurrence of SGA, LGA, and AGA fetuses did not differ between these groups. But, when evaluating a higher dose at 1000 mg/kg group, an increase in the frequency of SGA was observed in the control group even though there was no change in placental weight, placental index, and placental efficiency.

Reduced placental weight can be associated with fetal malnutrition, reduced growth, low weight gain, and lower placental index. The outcome of these changes can be reduced fetal weight [38]. Reduced fetal weight can also correlate to altered placental efficiency.

Assessments of placental function and the effects of gestational insults often use the ratio of birth weight to placental weight as a measure of placental efficiency [39]. This ratio has been suggested to reflect placental exchange surface area, nutrient transport rates, and blood flow, potentially reflecting placental development and function adjustments to meet fetal demand [40, 41]. This parameter, a variable between species, translates the maternal–fetal relationship established during the gestational period and is a determinant of intrauterine growth since it is responsible for the nutritional and hormonal supply of the fetus [42]. Therefore, placental efficiency is influenced by the size, morphology, blood flow, and transport efficiency that can occur by simple diffusion and active transport [43].

Regarding the occurrence of malformations, it was observed that EESl, at doses of 100 and 1,000 mg/kg, increased the frequency of external and skeletal malformations. However, visceral malformations increased only for the EESl (100 mg/kg) group. Among the external malformations, the ones that occurred the most were curved tail, hyperlordosis, and scoliosis, which are spinal alterations.

Giampietro et al. [44] report that congenital vertebral malformations in the human population are etiologically heterogeneous with poorly understood environmental and genetic factors. Their prevalence is estimated to be between 0.13–0.51/1,000 live births. Among congenital spinal malformations are kyphosis and scoliosis, for example. According to Wang et al. [45], congenital scoliosis is a curvature of the spine resulting from abnormal vertebral development, with an incidence in the general population of approximately 1/1,000 to 1/2,000, also resulting from environmental and genetic factors. Among the environmental factors, one can also consider the consumption of drugs and teas, for example. These clinical findings can also be reproduced in experimental models.

Li et al. [46] observed that vitamin A deficiency causes congenital vertebral defects in rats. In their study with rats, Welch et al. [47] demonstrated that anabasine, a nicotinic receptor agonist substance extracted from N. glauca, leads to higher numbers of fetuses with scoliosis in the treated groups than in the control group. Medeiros et al. [48] and Gardner et al. [49] used a rat model to study congenital disabilities caused by the plant Mimosa tenuiflora and its compounds and identified bone malformations, including scoliosis, lordosis, and microcephaly.

The primary visceral malformations identified were hydrocephalus and hydronephrosis. Hydrocephalus did not present significant differences between the groups. Therefore, they are considered variants of normality. Hydronephrosis increased significantly in the EESl (100 mg/kg) group. However, these changes may regress at birth since the fetuses were collected on gestational day 18, and the end of natural gestation would only occur on gestational day 21. Thus, these alterations could be correctable. However, this data requires attention.

Some studies in the field [27, 50] describe variants of normality as changes that may regress as gestation progresses or at birth since the fetuses would have completed their development.

Regarding skeletal malformations, it was observed that EESl increased the frequency of reduced ossification or absence of ossification, especially in the skull and sternum. This change may occur due to the early removal of the fetus (18^th^ gestational day). Thus these observations would be transient, with less impact on the survival or health of the individual. Delayed ossifications are therefore included in skeletal variations [51]. However, as there was a statistical increase in frequency, we infer that this data requires attention, although the procedure of early collection of fetuses is protocol and indicated by the specialized literature of the area [26, 52].

Evaluation of skeletal development is routinely performed in embryofetal development studies to support the development of new therapeutic agents because ossification patterns in humans and rodents are similar, with bones originating from intramembranous or endochondral ossification and processes controlled by genetic and environmental factors [53]. Therefore, this is a crucial endpoint to evaluate since the toxic effects of xenobiotics on fetal bone development can result in skeletal malformations, delayed skeletal ossification, and skeletal variants [54].

## Conclusions

Given the above, it is considered that the ethanolic extract of *Salvia lachnostachys* Benth is not maternotoxic and does not alter the reproductive performance of females according to the present design. However, it does change embryofetal development despite having low teratogenic potential. Thus, we don’t recommend using this extract during the gestational period.

## Data Availability

All data generated or analysed during this study are included in this published article.
